# Increase over time of antibody levels 3 months after a booster dose as an indication of better protection against Omicron infection

**DOI:** 10.1080/22221751.2023.2184176

**Published:** 2023-03-09

**Authors:** Rea Bingula, Hélène Chabrolles, Benjamin Bonnet, Christine Archimbaud, Amélie Brebion, Justine Cosme, Amandine Ollier, Frédéric Dutheil, Maud Junda, Audrey Mirand, Christel Regagnon, Magali Vidal, Cécile Henquell, Bertrand Evrard

**Affiliations:** a UMR UNH, ECREIN, Immunology Laboratory, Faculty of Medicine, Clermont Auvergne University, Clermont-Ferrand, France; b Virology Department, Clermont-Ferrand University Hospital (CHU Clermont-Ferrand), 3IHP, Clermont-Ferrand, France; c CNRS UMR 6023, LMGE, Clermont Auvergne University, Clermont-Ferrand, France; d Immunology Department, Clermont-Ferrand University Hospital (CHU Clermont Ferrand), Clermont-Ferrand, France; e Clinical Research and Innovation Direction, Clermont-Ferrand University Hospital (CHU Clermont-Ferrand) 3 IHP, Clermont-Ferrand, France; f Preventive and Occupational Medicine, Clermont-Ferrand University Hospital (CHU Clermont-Ferrand), Clermont-Ferrand, France; g CNRS, LaPSCo Physiological and Psychosocial Stress, Clermont Auvergne University, Clermont-Ferrand, France

**Keywords:** SARS-CoV-2, humoral and cellular response, anti-RBD IgG, IFN-γ, mRNA third booster dose

## Abstract

The third, “booster”, vaccination increases the overall immune response against SARS-CoV-2 variants. However, after the initial peak at around 3 weeks post-vaccination, anti-spike antibody levels decline. Post-booster kinetics of cellular response has been less investigated and there is no documented evidence of a true boosting effect. Furthermore, multiple studies underline the less effective immune responses against Omicron, the latest variant of concern, at both humoral and cellular levels. In this letter, we analyse humoral (anti-RBD IgG levels) and cellular (IFN-γ release assay) immune response in 205 health care workers 3 weeks and 3 months after administration of an mRNA-based booster dose, either mRNA-1273 or BNT162b2. Since all subjects were SARS-CoV-2 infection-naïve, we also looked at the incidence of Omicron infection between 3 and 6 months post-booster.

At both timepoints, 3x mRNA-1273 vaccination had the highest overall antibody and IFN-γ levels, followed by 3x BNT162b2 vaccination and heterologous mRNA-based regimens. Heterologous ChAdOx1–mRNA-based regimen had the lowest antibody levels while cellular response equal to that of 3x BNT162b2 vaccination and heterologous mRNA-based regimens. Our results show that both humoral and cellular responses waned at 3 months for all vaccination regimens. However, we identified three trajectories of dosage variation. Interestingly, the subgroup of subjects with increasing anti-RBD IgG levels over time had a lower incidence of Omicron infection. Whether increasing humoral response at 3 months post-booster is more indicative of protection than a high initial peak remains to be confirmed in a larger cohort.

## Dear Editor,

Real-life data showing the impact of an mRNA booster dose against COVID-19, independently of primary vaccination, evidenced significantly increased cellular and humoral immune responses immediately after administration [1]. Other studies following the kinetics of antibody production in subjects’ sera after a booster found a peak at around 3 weeks, which declined thereafter [2,3]. To our best knowledge, there are only a few documented reports of cellular response, ranging mostly from 4 to 14 weeks post mRNA-based booster. They reported no real boosting effect and/or decrease in time in IFN-γ secretion (ELISpot) upon challenge against wild-type virus [2,4].

An ever-growing number of studies have described less effective immune responses against Omicron, the last SARS-CoV-2 variant of concern that emerged in South Africa on November 2021, than against previous variants [5,6]. After an mRNA-based booster, the humoral anti-Omicron response in never-infected fully vaccinated subjects [7,8] seems to improve. However, the neutralization capacity of the antibodies is less than that of other variants [2,9] and no “reliable” IgG antibody protection limit has been determined [10]. With regard to cellular response, even lower IFN-γ production against Omicron antigens has been reported compared to that against other variants [4,6].

In this letter, we analyse both humoral and cellular immune response 3 weeks and 3 months after administration of an mRNA booster dose to 205 health care workers (HCWs) in the COVIDIM prospective longitudinal cohort with no record of previous SARS-CoV-2 infection up to 3 months following the booster. The HCWs were also monitored for SARS-CoV-2 infection between 3 and 6 months post-booster, the period coinciding with the Omicron wave. The aim was to assess the variation of both humoral and cellular immune response between these timepoints and whether the variation could be related to the occurrence of SARS-CoV-2 infection in the following months.

Participants received their booster dose from September 2021 to March 2022. Depending on their vaccination scheme, they were divided into four groups: three vaccine doses of BNT162b2 (Pfizer BioNTech) (“3x BNT162b2”); three vaccine doses of mRNA-1273 (Moderna) (“3x mRNA-1273”); a heterologous protocol of mRNA-based vaccines (“3x RNA”); and a heterologous protocol of ChAdOx1 (Oxford, AstraZeneca) and mRNA-1273 and/or BNT162b2 (“Mix ChAdOx1–RNA”). Group characteristics are given in Table S1. Natural SARS-CoV-2 infection was defined as anti-nucleocapsid positivity (cut-off index value [optical density]: 1.4, Architect SARS-CoV-2 IgG assay, Abbott) and/or history of positive PCR result on nasopharyngeal swab. SARS-CoV-2 anti-RBD IgG response was quantified by Architect SARS-CoV-2 IgG II Quant assay (Abbott). Memory T-cell response to SARS-CoV-2 spike antigens was quantified by interferon-gamma (IFN-γ) release assay (QuantiFERON SARS-CoV-2, Qiagen). Results for Ag.1 and Ag.2 were closely similar, so we opted to give only Ag.2 data (stimulation of both CD4^+^ and CD8^+^ T cells).

**Figure 1.**
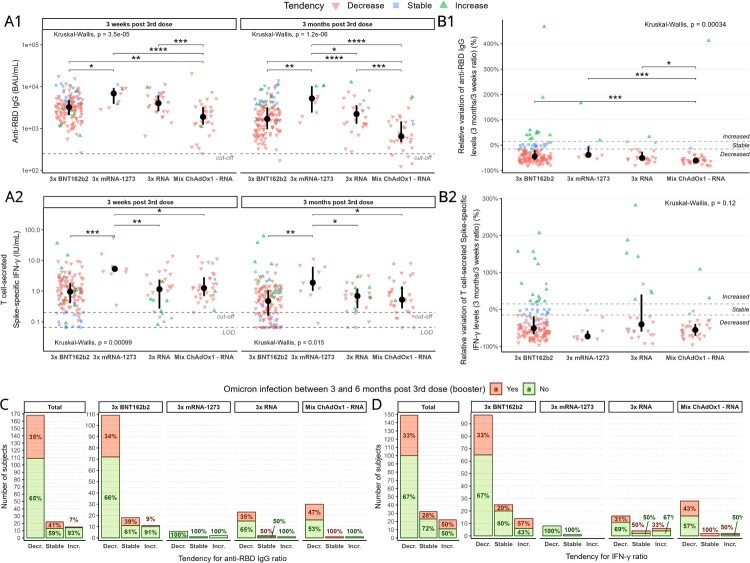
Humoral and cellular immune responses. Levels of **A1** anti-RBD IgG (positivity cut-off = 250 BAU/mL) and **A2 **T cell-secreted Spike-specific IFN-γ (positivity cut-off = 0.2 IU/mL, LOD = 0.065 IU/mL) at 3 weeks and 3 months post booster dose. Values found under LOD have been substituted by LOD. Relative variation of levels (3 months/3 weeks ratio) for **B1** anti-RBD IgG and **B2 **T cell-secreted Spike-specific IFN-γ. Stability range was determined as ±15% variance (within-laboratory precision) between timepoints (plus delta < 0.1 UI/mL for cellular response). Data were analysed with Kruskal-Wallis test followed by Dunn’s pairwise comparison without correction, if significant. The incidence of infection with Omicron according to tendency group for **C** anti-RBD IgG and **D** T cell-secreted Spike-specific IFN-γ. Difference in group composition was evaluated by odds-ratio test, followed by Fisher’s exact test. *p < 0.05; **p < 0.01; ***p < 0.001; ****p < 0.0001; BAU – binding antibody unit; IU – international unit; LOD – limit of detection.

Overall data show that at both timepoints anti-RBD IgG levels (hereafter “Ab”) expressed in binding antibody units (BAU)/mL were significantly the lowest for Mix ChAdOx1–RNA (Figure 1(A1), Table S1). Mix ChAdOx1–RNA also had the greatest relative decrease in Ab levels between timepoints (median: −60.7%), which was significantly different from that of all other groups (Figure 1(B1)). Group 3x mRNA-1273 had significantly the highest Ab levels both at 3 weeks (except vs 3x RNA) and at 3 months (Figure 1(A1)), while 3x BNT162b2 and 3x RNA had similar Ab levels. In addition, 3x mRNA-1273 had the lowest, albeit non-significant, relative decrease in time of all groups (Figure 1(B1)).

Secreted IFN-γ levels were significantly highest for 3x mRNA-1273 (Figure 1(A2)). The other three groups had a similar value at both time points. Despite maintaining the absolute highest response (Figure 1(A2)), 3x mRNA-1273 showed the greatest, albeit non-significant, relative decrease over time (median: −72.7%) of all groups (Figure 1(B2)).

Based on relative variation between timepoints in Ab and IFN-γ levels we identified three tendency subgroups for each analysis (Figure 1(B1,B2)): subjects with either stable (Ab_Stb or IFN_Stb), increased (Ab_Inc or IFN_Inc) or decreased (Ab_Dec or IFN_Dec) levels over time (Table S1). Importantly, the tendency for humoral and cellular responses differed within subjects. The Ab levels (Figure 1(A1)) were highest at 3 weeks for Ab_Stb subjects (vs Ab_Dec *p* < 0.001, vs Ab_Inc *p* = 0.002 for 3x BNT162b2, the same profile observed in the other groups but non-significant). However, at 3 months Ab_Inc subjects had similar Ab levels to those of Ab_Stb subjects, which were both higher than those of Ab_Dec subjects (*p* < 0.001 for 3x BNT162b2, the same profile as in the other groups). Conversely, for IFN-γ levels (Figure 1(A2)), the lowest levels at both timepoints were those of IFN_Stb subjects (significant for 3x BNT162b2 and 3x RNA), with 60% of points being below cut-off. There was no difference in levels between IFN_Inc and IFN_Dec subjects except for 3x BNT162b2 at 3 months, which had higher levels in IFN_Inc subjects (*p* < 0.0001).

Finally, we looked at the incidence of Omicron infection between 3 and 6 months post mRNA booster in each tendency subgroup and/or per vaccination strategy. First, there was no significant difference in levels or relative variation for either Ab or IFN-γ between Omicron-infected and infection-naïve subjects whatever the vaccination scheme (Figure S1). Nobody in the 3x mRNA-1273 group was infected. The incidence of Omicron infection was lowest in Ab_Inc subjects (Figure 1(C)), and significant in the overall cohort (odds-ratio estimate vs Ab_Dec: 6.66, Fisher-exact test 0.023) (Figure 1(C)). In contrast, no significant link between infection incidence and variation tendency for IFN-γ was noted (Figure 1(D)).

We observed an overall waning of the immune response at 3 months in all vaccination schemes (as previously reported [2–4]), with 3x mRNA-1273 conserving the highest humoral and cellular responses. This observation confirms a general decrease in cellular response at 3 months using a different technique to that used previously [2,6]. Overall, 3x BNT162b2 and 3x RNA showed similar results. In line with previous publications [1,7,11,12], heterologous ChAdOx1–mRNA-based 3-dose vaccination induced lower Ab levels than exclusively mRNA-based 3-dose vaccination (in both homologous and heterologous regimens) but a comparable cellular response to that of 3x BNT162b2 and 3x RNA. In contrast, Behrens et al. [13] observed similar Ab levels in heterologous ChAdOx1–BNT162b2-based 3-dose vaccination and 3x BNT162b2, an absence of difference possibly due to the unequal sampling intervals after the booster between the groups in their study. Our results are in line with those evidencing no “reliable” protection threshold against Omicron [10], probably due to very low cross-reactivity especially in vaccinated SARS-CoV-2 infection-naïve subjects despite a strong humoral and cellular response [9,14].

We are the first to show that increasing humoral response at 3 months post-booster in a subgroup of subjects could be indicative of better protection rather than a high initial peak, potentially due to improved B cell affinity maturation compared to immediate clonal expansion of existing memory [15]. This intriguing observation requires further investigation, but could indicate increased booster efficacy if it increases germinal centre maturation. The major limitations of the study are the small number of subjects within vaccine groups other than 3x BNT162b2, due to the French vaccination policy, and the general low incidence of subjects with a “non-decreasing” response. Our findings, therefore, need to be confirmed with a larger target sample.

## Supplementary Material

Supplemental Material

## Data Availability

The datasets generated during and/or analysed during the current study are available from the corresponding author on reasonable request.

## References

[1] Herzberg J, Fischer B, Becher H, et al. Cellular and humoral immune response to a third dose of BNT162b2 COVID-19 vaccine – a prospective observational study. Front Immunol. 2022;13:1–9. 10.3389/fimmu.2022.896151 PMC9286388 35844588

[2] Liu X, Munro APS, Feng S, et al. Persistence of immunogenicity after seven COVID-19 vaccines given as third dose boosters following two doses of ChAdOx1 nCov-19 or BNT162b2 in the UK: three month analyses of the COV-BOOST trial. J Infect. 2022;84:795–813. 35405168 10.1016/j.jinf.2022.04.018 PMC8993491

[3] Gilboa M, Regev-Yochay G, Mandelboim M, et al. Durability of immune response after COVID-19 booster vaccination and association with COVID-19 omicron infection. JAMA Netw Open. 2022;5:e2231778. 36107426 10.1001/jamanetworkopen.2022.31778 PMC9478782

[4] Maringer Y, Nelde A, Schroeder SM, et al. Durable spike-specific T-cell responses after different COVID-19 vaccination regimens are not further enhanced by booster vaccination. Sci Immunol. 2022;7:eadd3899. 36318037 10.1126/sciimmunol.add3899 PMC9798886

[5] Accorsi EK, Britton A, Fleming-Dutra KE, et al. Association between 3 doses of mRNA COVID-19 vaccine and symptomatic infection caused by the SARS-CoV-2 omicron and delta variants. JAMA. 2022;327:639. 35060999 10.1001/jama.2022.0470 PMC8848203

[6] Reynolds CJ, Pade C, Gibbons JM, et al. Immune boosting by B.1.1.529 (omicron) depends on previous SARS-CoV-2 exposure. Science (80-). 2022;377:eabq1841. 10.1126/science.abq1841 PMC9210451 35699621

[7] GeurtsvanKessel CH, Geers D, Schmitz KS, et al. Divergent SARS-CoV-2 omicron-reactive T and B cell responses in COVID-19 vaccine recipients. Sci Immunol. 2022;7:eabo2202. 35113647 10.1126/sciimmunol.abo2202 PMC8939771

[8] Wang X, Zhao X, Song J, et al. Homologous or heterologous booster of inactivated vaccine reduces SARS-CoV-2 omicron variant escape from neutralizing antibodies. Emerg Microbes Infect. 2022;11:477. 35034583 10.1080/22221751.2022.2030200 PMC8820826

[9] Miyamoto S, Arashiro T, Adachi Y, et al. Vaccination-infection interval determines cross-neutralization potency to SARS-CoV-2 omicron after breakthrough infection by other variants. Med. 2022;3:249–261.e4. 35261995 10.1016/j.medj.2022.02.006 PMC8894731

[10] Tré-Hardy M, Cupaiolo R, Wilmet A, et al. Assessment 2 months after the administration of a 3rd dose mRNA: a new variant-adapted vaccine is expected. J Infect. 2022;84:e31–e33. 10.1016/j.jinf.2022.02.009 PMC8847092 35181372

[11] Munro APSS, Janani L, Cornelius V, et al. Safety and immunogenicity of seven COVID-19 vaccines as a third dose (booster) following two doses of ChAdOx1 nCov-19 or BNT162b2 in the UK (COV-BOOST): a blinded, multicentre, randomised, controlled, phase 2 trial. Lancet. 2021;398:2258–2276. 34863358 10.1016/S0140-6736(21)02717-3 PMC8639161

[12] Atmar RL, Lyke KE, Deming ME, et al. Homologous and heterologous COVID-19 booster vaccinations. N Engl J Med. 2022;386:1046–1057. 35081293 10.1056/NEJMoa2116414 PMC8820244

[13] Behrens GMN, Barros-Martins J, Cossmann A, et al. BNT162b2-boosted immune responses six months after heterologous or homologous ChAdOx1nCoV-19/BNT162b2 vaccination against COVID-19. Nat Commun. 2022;13:1–10. 35982040 10.1038/s41467-022-32527-2 PMC9387891

[14] Jacobsen H, Cobos Jiménez V, Sitaras I, et al. Post-vaccination T cell immunity to omicron. Front Immunol. 2022;13:1–8. 10.3389/fimmu.2022.944713 PMC9386871 35990661

[15] Laidlaw BJ, Ellebedy AH. The germinal centre B cell response to SARS-CoV-2. Nat Rev Immunol. 2022;22:7–18. 34873279 10.1038/s41577-021-00657-1 PMC8647067

