# Supplementary material for: Increase over time of antibody levels 3 months after a booster dose as an indication of better protection against Omicron infection
Source: Emerg Microbes Infect. 2023 Mar 9;12(1):2184176. doi: 10.1080/22221751.2023.2184176 (PMC10013501; doi:10.1080/22221751.2023.2184176)
Supplement: Supplemental Material [file TEMI_A_2184176_SM5295.docx]

**Table S1.** Cohort characteristics

|  | 3x BNT162b2 | 3x mRNA-1273 | 3x RNA | Mix ChAdOx1–RNA | p-value |
| --- | --- | --- | --- | --- | --- |
| N | 138 | 9 | 26 | 32 |  |
| Age (years; median [IQR]) | 53 [46, 58] | 47 [43, 55] | 45 [35, 53] | 42 [36, 47] | **< 0.001^2^** |
| Female (%) | 84.8% | 88.9% | 80.8% | 75.0% | 0.58^1^ |
| Detailed vaccination scheme in chronological order of vaccination (N): |  |  |  |  |  |
| - 2x BNT162b2 - mRNA-1273 |  |  | 11 |  |  |
| - 2x mRNA-1273 - BNT162b2 |  |  | 15 |  |  |
| - ChAdOx1 - 2x BNT162b2 |  |  |  | 24 |  |
| - ChAdOx1 - BNT162b2 - mRNA-1273 |  |  |  | 4 |  |
| - 2x ChAdOx1 - BNT162b2 |  |  |  | 2 |  |
| - 2x ChAdOx1 - mRNA-1273 |  |  |  | 2 |  |
| Sampling intervals (days; median [IQR]) |  |  |  |  |  |
| - Booster: 3 weeks follow-up | 19 [16, 21] | 18 [15, 20] | 19 [15, 20] | 20 [16, 21] | 0.85^2^ |
| - Booster: 3 months follow-up | 84 [81, 91] | 87 [82, 89] | 85 [79, 90] | 83 [77, 92] | 0.69^2^ |
| Anti-RBD IgG |  |  |  |  |  |
| Levels (BAU/mL) (median [IQR]) |  |  |  |  |  |
| - At 3 weeks | 3236 [2083, 4783] | 6884 [3866, 9311] | 4048 [2594, 6229] | 1899 [1232, 3248] | **< 0.0001^2^** |
| - At 3 months | 1691 [961, 3228] | 5230 [2401, 10286] | 2259 [1292, 3618] | 654 [473, 1485] | **< 0.0001^2^** |
| Relative variation (3 months/3 weeks ratio) | -43.7 [-58,2, -20] | -38.2 [-43.8, -5.1] | -50.4 [-56.5, -27.2] | -60.7 [-67.9, -50.3] | **0.0003^2^** |
| Separation by variation tendency (N [freq]) |  |  |  |  |  |
| - Decrease | 109 (78,99 %) | 6 (66,67 %) | 23 (88,46 %) | 30 (93,75 %) |  |
| - Stable | 18 (13,04 %) | 1 (11,11 %) | 2 (7,69 %) | 1 (3,13 %) |  |
| - Increase | 11 (7,97 %) | 2 (22,22 %) | 1 (3,85 %) | 1 (3,13 %) |  |
| Spike-specific IFN-γ |  |  |  |  |  |
| Levels (IU/mL) (median [IQR]) |  |  |  |  |  |
| - At 3 weeks | 0.96 [0.41, 1.88] | 5.29 [4.27, 6.37] | 1.16 [0.29, 2.39] | 1.26 [0.69, 2.86] | **0.0010^2^** |
| - At 3 months | 0.48 [0.16, 1.07] | 1.88 [1.01, 6.21] | 0.69 [0.28, 1.22] | 0.53 [0.27, 1.42] | **0.015^2^** |
| Relative variation (3 months/3 weeks ratio) | -50.7 [-66.6, -18.5] | -72.7 [-79.8, -57.5] | -40.5 [-60.3, 40.6] | -55.4 [-71, -39] | 0.12**^2^** |
| Separation by variation tendency (N [freq]) |  |  |  |  |  |
| - Decrease | 97 (71,85 %) | 8 (88,89 %) | 16 (61,54 %) | 28 (87,5 %) |  |
| - Stable | 25 (18,52 %) | 1 (11,11 %) | 4 (15,38 %) | 2 (6,25 %) |  |
| - Increase | 13 (9,63 %) |  | 6 (23,08 %) | 2 (6,25 %) |  |
| ^1^ Fisher’s exact test; ^2^ Kruskal-Wallis test; BAU – binding antibody units; freq – frequency; IQR – interquartile range; IU – international unit; N – number of subjects | | | | | |

**
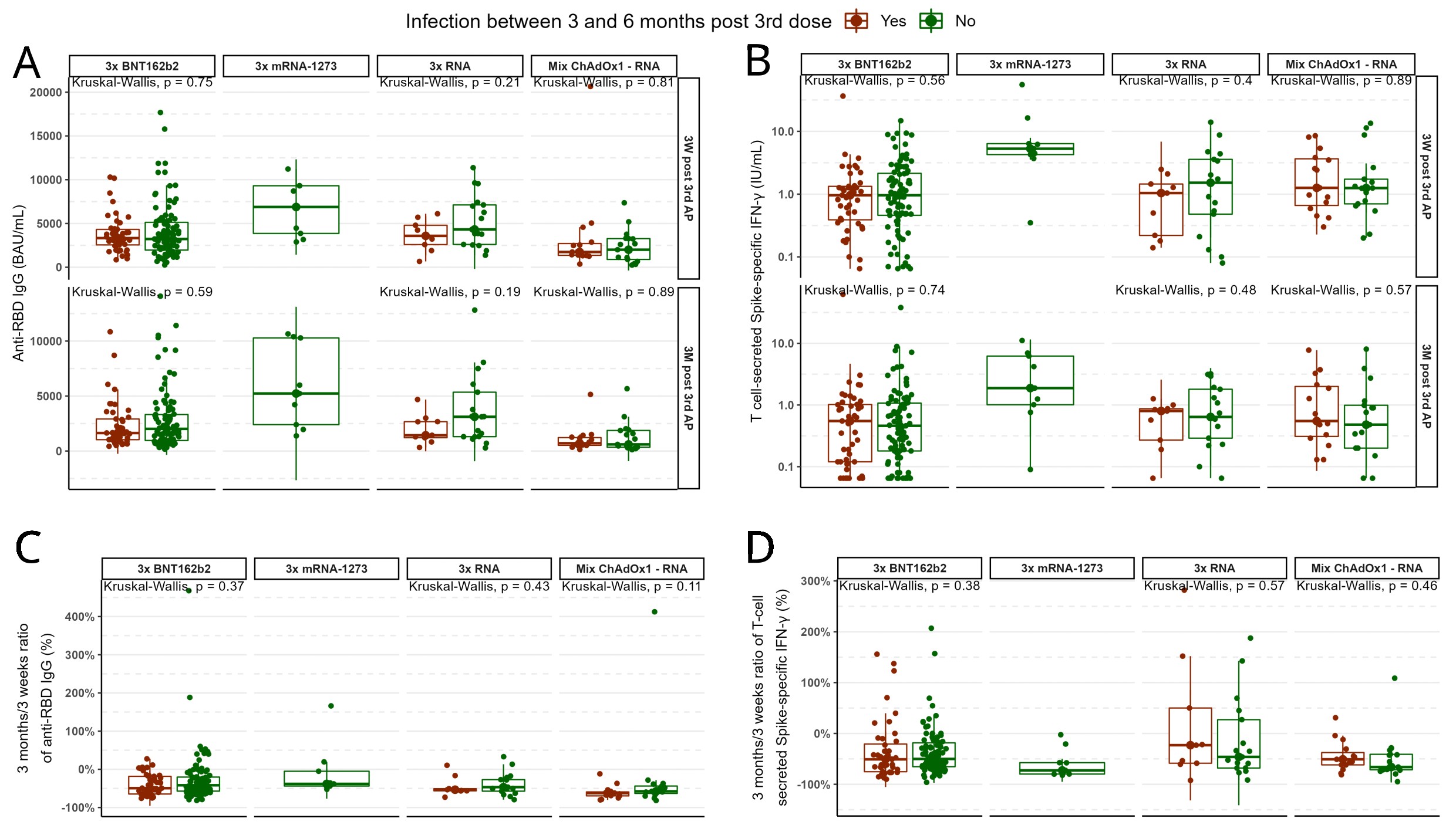
Figure S1.** Comparison of humoral and cellular responses at 3 weeks and 3 months between 3-to-6-months post-booster SARS-CoV-2 naïve and infected subjects. Levels of **A** anti-RBD IgG (positivity cut-off = 250 BAU/mL) and **B** T cell-secreted spike-specific IFN-γ (positivity cut-off = 0.2 IU/mL, LOD = 0.065 IU/mL) at 3 weeks and 3 months post-booster dose. Values found under LOD have been substituted by LOD. Relative variation of levels (3 months/3 weeks ratio) for **C** anti-RBD IgG and **D** T cell-secreted Spike-specific IFN-γ.
